# Glycine- and Alanine-Intercalated Layered Double Hydroxides as Highly Efficient Adsorbents for Phosphate with Kinetic Advantages

**DOI:** 10.3390/nano12040586

**Published:** 2022-02-09

**Authors:** Qian Zhang, Fangying Ji, Lei Jiang, Qiushi Shen, Yuanxiang Mao, Caocong Liu

**Affiliations:** College of Environment and Ecology, Chongqing University, Chongqing 400045, China; zhangqiancqu@cqu.edu.cn (Q.Z.); jginger067@gmail.com (L.J.); m15025439561@126.com (Q.S.); 20191701572@cqu.edu.cn (Y.M.); 20201701029@cqu.edu.cn (C.L.)

**Keywords:** layered double hydroxides, glycine, alanine, phosphate removal, adsorption kinetics, adsorption mechanism

## Abstract

Phosphate is the main cause of eutrophication. Layered double hydroxides (LDH) are considered to be promising phosphate adsorbents due to their high affinity and large capacity. In this study, we partially intercalated zwitterionic glycine and alanine into Cl-LDH (corresponding to MgAl-LDH with interlayer anion Cl^−^) and synthesized efficient inorganic–organic nanohybrids for phosphate removal with kinetic advantages. Gly-Cl-LDH, Ala-Cl-LDH and Cl-LDH were characterized, and their phosphate adsorption performances under the influence of environment factors (e.g., solution pH, coexisting anions, contact time and phosphate concentration) were investigated. The results show that Gly-Cl-LDH and Ala-Cl-LDH had larger specific surface areas and larger interlayer spaces than Cl-LDH, and exhibited better adsorption performance at a lower pH and better adsorption selectivity against SO_4_^2−^. Kinetic experiments indicated that Gly-Cl-LDH and Ala-Cl-LDH can reduce phosphate concentrations to a lower level in a shorter time. The pseudo-second-order kinetic constants of Gly-Cl-LDH and Ala-Cl-LDH were 1.27 times and 3.17 times of Cl-LDH, respectively (R^2^ > 0.996). The maximum adsorption capacities derived from a Langmuir model of Cl-LDH, Gly-Cl-LDH and Ala-Cl-LDH are 63.2 mg-P/L, 55.8 mg-P/L and 58.2 mg-P/L, respectively, which showed superiority over the prevailing phosphate adsorbents. This research provides highly efficient adsorbents for removing phosphate from aqueous solutions.

## 1. Introduction

Phosphate is an irreplaceable raw material in the agrochemical, food and household chemical industries [1], but excessive phosphate discharged into water bodies is a major source of eutrophication. As a target pollutant in wastewater treatment, phosphate could be removed through biological, physical and chemical techniques, including traditional biological methods, chemical precipitation, flocculation and membrane separation, etc. To meet increasingly stringent discharge regulations, the adsorption method has attracted attention due to its low infrastructure cost, the simplicity of its operational design, its elimination from concentrated and diluted solutions, its low sludge yield, its low energy consumption and its phosphate recovery potentiality [2,3]. The development of cost-efficient adsorbents with large adsorption capacities, high uptake rates and high selectivity is of significance and in demand. Extensively reported upon phosphate adsorbents include natural clay minerals [4,5], aluminum, calcium and iron-rich industrial waste residues [6], metal oxides and hydroxides [7], functionalized mesoporous silica [8], metal organic frameworks [9,10], etc. In recent decades, the study of phosphate adsorbents has mainly focused on the development of technologies such as modifying natural materials, preparing adsorbents from industrial waste residues and developing artificial functional adsorbents. Among them, the main purpose of material modification is to increase the phosphate adsorption capacity, kinetics and selectivity.

Layered double hydroxides (LDHs), also known as hydrotalcite-like compounds or anionic clays, are a series of anion exchange nanomaterials. LDHs have high affinity for orthophosphate [11], and exhibit superior adsorption capacity among the various researched phosphate adsorbents [2]. The chemical composition of LDH is ${[M}_{1-x}^{2+}M_{x}^{3+}{\left(\mathrm{OH}\right)}_{2}{][A}^{n-}{]}_{x/n}\cdot {\mathrm{yH}}_{2}O$, where M^2+^ and M^3+^ are metal cations, A^n−^ represents the interlayered anions and x is usually between 0.17 and 0.33 [12,13]. Although LDHs exist as natural minerals (e.g., hydrotalcite, stichtite, reevesite, etc.), LDHs with high purity, single phase and high crystallinity are also simple and economical to prepare in the laboratory [14]. In the past decades, the number of reported studies on LDH modification, which aim to further improve the morphology and structure [15], separating property [16], adsorption capacity [17] and adsorption selectivity [18], keeps rising.

The strong chemical bond within the hydroxide layer, and the weak bonds between the layers and interlayer anions, provide LDHs with their intrinsic anion exchange abilities. Thus, various functional guest molecules can enter the interlayer space of LDHs, and modify LDHs with desired functions without structural change [19,20]. The functional intercalation can adjust the interlayer space [21,22], reassemble the distribution of interlayer anions and further modify the adsorption properties of LDHs as needed. The intercalation guest anions include inorganic anions [2], organic anions [23], complex anions [24], isopolyacid and heteropolyacid anions [25], bioactive substances [26], etc. In the appropriate pH range, zwitterionic glycine and alanine can intercalate into LDH to reshape the interlayer space and enhance the electrostatic interaction with phosphate due to its zwitterionic nature. Therefore, the integration of LDH and glycine (or alanine) may result in novel adsorbents for phosphate. Only a few studies have considered amino acid-intercalated LDH hybrid nanomaterials as adsorbents for contaminants, such as heavy metal ions [27,28,29]. Studies on the effect of amino acid intercalation on the adsorption performance of LDHs, especially their adsorption selectivity and kinetics, are still lacking.

The aim of the present study was to study the effect of glycine intercalation on the structure, composition and phosphate adsorption performance of LDHs. Herein, we synthesized both the pristine LDH (Cl-LDH), the glycine-intercalated LDH (Gly-Cl-LDH) and the alanine-intercalated LDH (Ala-Cl-LDH), and characterized them via FESEM, specific surface area and pore size analysis, ICP-OES, TOC-TN analysis, PXRD, FTIR and zeta potential analysis. We subsequently studied the phosphate removal performances of Gly-Cl-LDH, Ala-Cl-LDH and Cl-LDH, including the influence of environmental factors (e.g., solution pH and coexisting anions), the adsorption kinetics and isotherms. Finally, we deduced the suggested adsorption mechanism based on the experimental data and characterization before and after phosphate uptake.

## 2. Materials and Methods

### 2.1. Materials

Analytical grade chemicals (MgCl_2_·6H_2_O, AlCl_3_·6H_2_O, NaOH, NaCl, NaNO_3_, Na_2_CO_3_, Na_2_SO_4_, HCl), a biochemical reagent (BR) and glycine and alanine (≥99.0%) were purchased from Kelong Chemical (Chengdu, China). Analytical grade KH_2_PO_4_ was purchased from Chuandong Chemical (Chongqing, China). Phosphate working solutions were freshly obtained from the stock solution (500 mg/L as element P). The pH of these was adjusted to the desired value with a negligible volume of diluted NaOH and HCl solutions. Ultrapure water (18.2 MΩ·cm) was used to prepare solutions, wash the coprecipitated products and rinse the experimental equipment.

### 2.2. Preparation of Cl-LDH, Gly-Cl-LDH and Ala-Cl-LDH

The preparation of each LDH included four sets of samples prepared under the same conditions, and the as-synthesized LDHs were well mixed for subsequent characterization and adsorption experiments. Cl-LDH was prepared with a fast coprecipitation method [2,30]. Briefly, 100 mL of solution containing MgCl_2_·6H_2_O and AlCl_3_·6H_2_O (the Mg/Al molar ratio was 2.0) and 100 mL of NaOH solution were simultaneously dropwise added into 100 mL ultrapure water by a BT100-2J peristaltic pump (Longer Pump, Baoding, China) at a speed of 4 mL/min. The dosage of NaOH was twice the sum of the moles of Mg and Al. The coprecipitation was carried out in a 40 °C water bath with mechanical agitation (300 rpm) using a DJIC120 agitator (Jintan Instruments, Changzhou, China). The pH of the mixture was controlled at ~10.0, which was continuously measured using a PHS-3C pH meter (Leici Instruments, Shanghai, China). The gelatinous precipitate was aged at 85 °C for 18 h, then centrifuged at 4000 rpm for 5 min and washed for four times using a TDZ5-WS centrifuge (Xiangyi Instruments, Changsha, China). After that, the solid products were dried at 105 °C for 24 h, ground with an agate mortar, sieved through a 200-mesh sieve and stored in a desiccator.

Glycine- and alanine-intercalated LDH (Gly-Cl-LDH and Ala-Cl-LDH) were prepared through a modified coprecipitation method, which is the same as the preparation method of Cl-LDH, except for the composition of precursors. A solution (100 mL) containing MgCl_2_·6H_2_O, AlCl_3_·6H_2_O and glycine (or alanine) at a molar ratio of 1.0: 2.0: 1.5, and another solution (100 mL) containing NaOH (the amount was twice the sum of the moles of Mg and Al) were respectively prepared. Under mechanical agitation, the two solutions were simultaneously added dropwise into 100 mL ultrapure water by a peristaltic pump at a speed of 4 mL/min. The coprecipitation was conducted in a 40 °C water bath, and the pH was maintained in the range of 9.0–10.0. After coprecipitation, the slurry was aged, centrifuged, washed, dried, ground, sieved and stored through the same method mentioned in the Cl-LDH synthesis.

### 2.3. Characterization of Cl-LDH, Gly-Cl-LDH and Ala-Cl-LDH

The morphology and chemical composition of Cl-LDH, Gly-Cl-LDH and Ala-Cl-LDH were revealed with a JSM-7800F field emission scanning electron microscope (FESEM) equipped with an energy dispersive spectrometer (EDS) system (JEOL, Tokyo, Japan). The particle size of each LDH was determined with a Mastersizer 2000 laser particle sizer (Malvern, UK). The specific surface areas and pore structures of the LDHs were measured using a Quadrasorb 2MP specific surface and pore size analyzer (Quantachrome, Boynton Beach, FL, USA). Samples were degassed under vacuum at 80 °C overnight. The contents of Mg, Al and P in LDH were quantitatively analyzed using an iCAP 6300 Duo inductively coupled plasma optical emission spectrometer (Thermo Scientific, Waltham, MA, USA) after dissolving the powder samples in 0.1 M HNO_3_ solutions. The contents of C and N were determined with a TOC-V_CPH_ total organic carbon analyzer (Shimadzu, Kyoto, Japan). Both the ICP-OES and TOC-TN analyses were conducted in triplicate, and results were reported as a mean and standard deviation. The limit of detection and quantification of ICP-OES and TOC-TN are detailed in Section S4 of the Supplementary Materials. The powder X-ray diffraction (PXRD) analysis was conducted on a D/max-1200 X-ray diffractometer (Rigaku, Tokyo, Japan) with a Ni-filtered CuK_α_ radiation (λ = 0.15418 nm). Data were collected in the 2θ range of 5~75° with an angular step of 0.02° at a scanning speed of 2.5°/s. The Fourier transform infrared (FTIR) spectra were recorded with an IR Prestige-21 infrared spectrophotometer (Shimadzu, Kyoto, Japan) using the KBr pellet method. The zeta potential of each LDH was analyzed by a Zetasizer Nano ZS90 nanoparticle size and zeta potential analyzer (Malvern, UK). The LDHs after phosphate adsorption, including P-Cl-LDH, P-Gly-Cl-LDH and P-Ala-Cl-LDH, were obtained by centrifugation, and washed with ultrapure water more than 2 times. For the preservation of their morphology, P-LDH was freeze-dried for 48 h and then characterized by SEM. Other characterizations were performed on 105 °C blast-dried P-LDH.

### 2.4. Adsorption Experiments

Phosphate adsorption experiments were conducted in batch mode in capped serum bottles containing 100 mL phosphate solution under constant magnetic agitation (120 rpm) at room temperature (25 ± 0.5 °C) and replicated three times. Filtrated liquid samples were collected by a syringe filter (0.22 μm).

The effect of solution pH was investigated by adding 0.03 g (0.3 g/L) LDH into 100 mL of 20 mg P/L phosphate solution, and the initial pH was adjusted to 2.0–11.0. The retention time was 2 h, which was determined in the preliminary experiment. To investigate the phosphate selectivity of LDH, artificial wastewater solutions were prepared by mixing KH_2_PO_4_ with NaCl, NaNO_3_, Na_2_CO_3_ or Na_2_SO_4_ and adjusting the pH of the hybrid to 6.50 ± 0.02. Each of the mixed solutions (100 mL) contained 0.1 mmol of phosphate and 0.1 mmol of the coexisting anion (Cl^−^, NO_3_^−^, HCO_3_^−^ or SO_4_^2−^). The adsorption experiment was conducted by adding 0.5 g/L LDH and reacting for 2 h.

For the kinetics study, 0.05 g LDH was added into 100 mL of 20 mg P/L phosphate solution at pH~5.4 without adjustment. The supernatant was collected at different time intervals up to 2 h to ensure the attainment of the adsorption equilibrium.

The adsorption isotherm was investigated by adjusting the initial phosphate concentrations from 5 mg P/L to 100 mg P/L. The initial pH was adjusted to 6.50 ± 0.02. The dosage of LDH was 0.3 g/L, and the retention time was 2 h.

The concentration of phosphate (as total P) was measured through the Molybdenum Blue Method on a UVmini-1240 UV-vis spectrophotometer (Shimadzu, Kyoto, Japan) at the wavelength of 700 nm. The limit of detection and quantification of this method are detailed in Section S4 of the Supplementary Materials.

### 2.5. Data Analyses

The calculation of parameters describing the adsorption performance, including phosphate removal capacity at equilibrium (*q_e_*, mg/g), phosphate removal efficiency (*R*) and distribution coefficient (*K*_d_), are elaborated in Section S1 in the Supplementary Materials. Results are displayed as mean ± standard deviation (*n* = 3). A paired *t*-test was performed to determine the significant differences among the groups. The observed differences were statistically significant when *p* < 0.05. The kinetics data were fitted to a pseudo-first model, pseudo-second model, Elovich model, Avrami model and intrinsic model, and the isotherm data were fitted with four two-parameter models, including the Langmuir, Freundlich, Temkin and Dubinin–Radushkevich (D-R) isotherm models, as elaborated in Section S2 of the Supplementary Materials. All the fittings were conducted with non-linear regression on OriginPro software. The kinetic data in some references are obtained through Plot Digitizer software.

## 3. Results and Discussions

### 3.1. Characterization of LDH

#### 3.1.1. FESEM Images and Surface Properties

**Figure 1:**
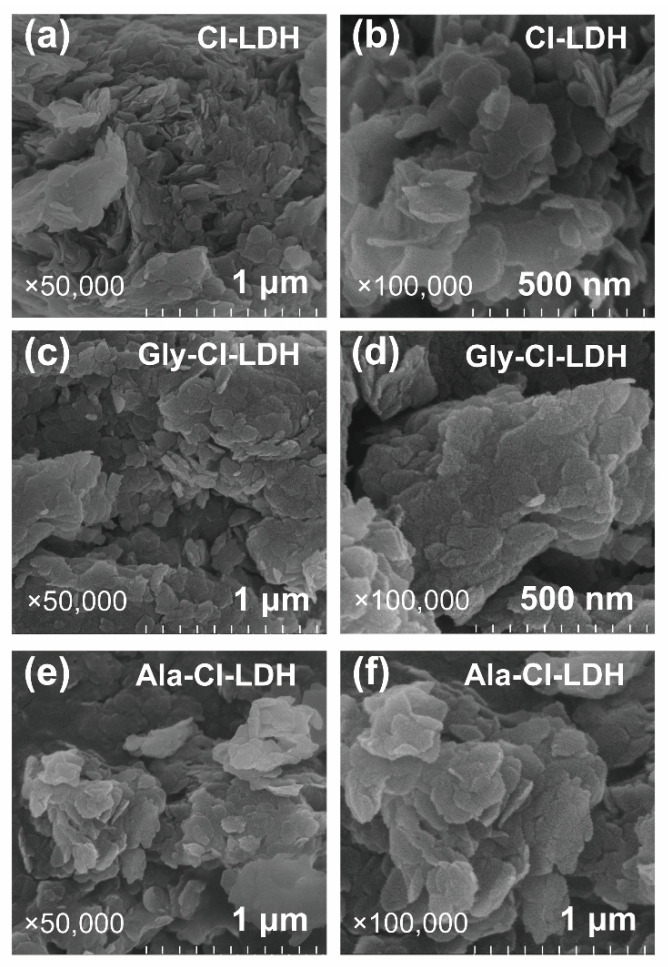
SEM images of Cl-LDH (**a**,**b**), Gly-Cl-LDH (**c**,**d**) and Ala-Cl-LDH (**e**,**f**).

In the FESEM images of Cl-LDH (Figure 1a,b), Gly-Cl-LDH (Figure 1c,d) and Ala-Cl-LDH (Figure 1e,f), the three LDHs exhibit typical lamellar morphology and a non-uniform irregular aggregation status. The plate-like morphology is typical for LDH materials. The aggregation status is also related to the synthesis method, including the coprecipitation, drying and sieving processes. The particle distribution of the as-synthesized LDHs is shown in Figure S4 and Table S1. After being dispersed in water, the *d*_(0.5)_ of Cl-LDH, Gly-Cl-LDH and Ala-Cl-LDH was 30.96, 30.63 and 27.31 μm, respectively. Due to the same drying and grinding process, the particle size and distribution of the three LDHs are similar. The particle size of Ala-Cl-LDH and Gly-Cl-LDH are slightly smaller than Cl-LDH. According to the N_2_ adsorption–desorption isotherm curves of Cl-LDH, Gly-Cl-LDH and Ala-Cl-LDH (Figure S5), the three isotherm curves are classified as type IV isotherms with the H3-type hysteresis loop. The findings confirm that the as-synthesized adsorbents have slit-shaped pores formed by the stacking of plate-like materials, which is in accordance with other reported LDH materials [31,32]. Through the calculation based on the Brunauer–Emmett–Teller (BET) method, the specific surface areas of Cl-LDH, Gly-Cl-LDH and Ala-Cl-LDH are 13.4, 19.0 and 21.5 m^2^/g, respectively. The specific surface area of Gly-Cl-LDH and Ala-Cl-LDH are slightly larger than that of CL-LDH. Generally, the increase in the porosity of LDH facilitates the exposure of active adsorption sites. However, due to the hard agglomeration of the adsorbent caused by the drying process, the specific surface area and pore structure were also affected by the grinding process.

#### 3.1.2. Chemical Composition Analysis

Based on the results of the ICP-OES and TOC-TN analyses and the charge balance considerations, we speculated the chemical composition formulas of Cl-LDH and Gly-Cl-LDH (Table 1). The Mg/Al molar ratio of Cl-LDH, Gly-Cl-LDH and Ala-Cl-LDH is 2.0, which is in accordance with the values in the precursors. The findings also demonstrate that the amount of glycine and alanine intercalated into LDH is much lower than that in the precursor. Glycine or alanine accounts for ~10% of the interlayer anions. The partial intercalation of glycine could be due to the competition between glycine/alanine and the widely presented Cl^-^ in the coprecipitation reaction system.

#### 3.1.3. PXRD Analysis

**Figure 2:**
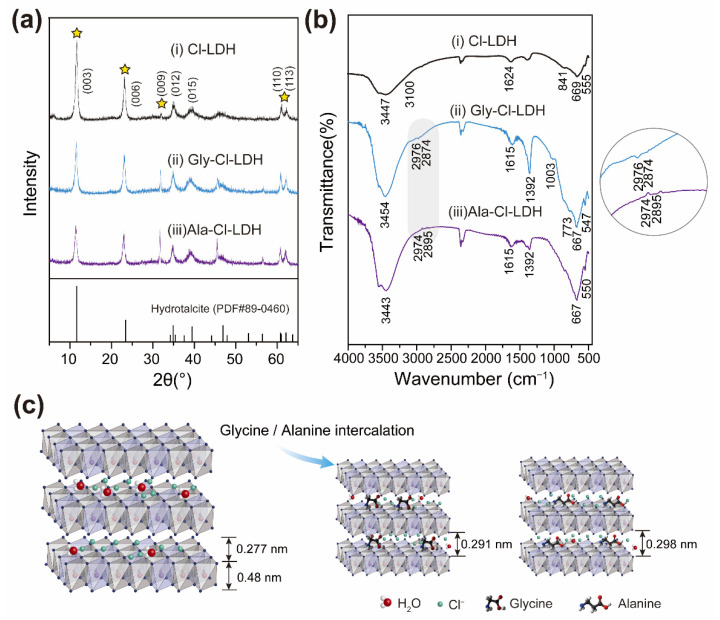
(**a**) XRD patterns (characteristic reflections are marked with stars) and (**b**) FTIR spectra of Cl-LDH and Gly-Cl-LDH; (**c**) is the schematic structural models of Cl-LDH, Gly-Cl-LDH and Ala-Cl-LDH.

In Figure 2a, the characteristic reflections, including (003), (006), (009), (012), (015), (018), (110) and (113), confirm the basic structure of the as-synthesized LDHs [15,28,33]. The high intensity of the main reflections, (003), (006) and (009), indicates the high crystallinity of the samples. Lattice constants *d*_003_ and *d*_110_ were calculated from the position and half-width of reflections (003) and (110) using the Bragg diffraction formula, and are presented in Table 2. The interlayer space, which reveals the stacking law of the hexagonal crystal lattice, was calculated by subtracting the thickness of the lattice (~0.48 nm) [14] from *d*_003_. The interlayer spaces of Cl-LDH, Gly-Cl-LDH and Ala-Cl-LDH are 2.77, 2.91 and 2.98 Å, respectively. The interlayer space of Gly-Cl-LDH and Ala-Cl-LDH is slightly higher than that of the pristine Cl-LDH, indicating that the intercalation of the functional guest influences the interlayer space of LDH. The interlayer space of Ala-Cl-LDH is larger than that of Gly-Cl-LDH, which also shows that the interlayer spacing is related to the size of the interlayer anions. In view of the size of the amino acid molecule, glycine and alanine are intercalated in a near-parallel orientation and a monolayer arrangement, which is attributed to the absence of large hydrophobic groups in the chemical composition [34]. The orientation and arrangement are in accordance with the findings of [34,35]. The horizontal arrangement of the interlayer amino acids was also observed when LDH was intercalated with phenylalanine, serine and tyrosine [29]. The crystallographic parameter *a*, which corresponds to the cation–cation distance and reveals the cations dispersion, was calculated from the lattice constant *d*_110_ (a = 2 *d*_110_) [36]. In Figure 2a, the *a* values of Cl-LDH, Gly-Cl-LDH and Ala-Cl-LDH are 3.03, 3.05 and 3.05 Å, confirming the consistency of the Mg/Al molar ratio in the chemical composition. Figure 2c shows the schematic structural models of Cl-LDH, Gly-Cl-LDH and Ala-Cl-LDH. 

#### 3.1.4. FTIR Spectroscopy

Figure 2b shows the characteristic FTIR vibrations of the octahedral sheet and the interlayer glycine or alanine. In the FTIR spectra of Cl-LDH, Gly-Cl-LDH and Ala-Cl-LDH, the broad bands at 3400 to 3500 cm^−1^, with a shoulder around 3100 cm^−1^, are attributed to the H-bonding stretching vibrations of the OH group (ν_OH_) in the octahedral lattice, interlayer water and physically adsorbed water molecules [29]. The shoulder present around 3100 cm^−1^ can be attributed to the water molecules hydrogen bonded to the carbonate ions (H_2_O–CO_3_^2−^ bridging mode) present in the interlayer region of LDH, which involves mostly hydrogen bonds [29]. The sharp bands at 1624 cm^−1^ (Figure 2b(i)) and 1615 cm^−1^ (Figure 2b(ii,iii)) are associated with the bending vibration of the interlayer water molecules ($\delta_{H-O-H}$). The bands in the lower wavenumber region (<850 cm^−1^) originate from the M-O and O-M-O (M is Mg or Al) lattice vibration modes. New bands in the region of 1300 to 3000 cm^−1^ were observed in the FTIR spectrum of Gly-Cl-LDH (Figure 2b(ii)) and Ala-Cl-LDH (Figure 2b(iii)), indicating the presence of glycine or alanine in the interlayer space of LDH. These absorption bands are relatively weak due to the limited amount of intercalated glycine or alanine. Specifically, the bands centered at 2976 cm^−1^ (Figure 2b(ii)) and 2974 cm^−1^ (Figure 2b(iii)) come from the alkyl C-H stretching vibration (ν_C-H_); the bands at 2874 cm^−1^ (Figure 2b(ii)) and 2895 cm^−1^ (Figure 2b(iii)) are due to the amine N-H stretching (ν_N-H_); the bands at 1392 cm^−1^ correspond to the symmetric stretching vibration of R-COO^-^ ($\nu_{{R-\mathrm{COO}}^{-}}$). The bands of CO_3_^2−^ were found in the FTIR spectra of the three LDHs, which may be due to the introduction of small amounts of CO_2_ from the air during the preparation process. This finding is consistent with the TOC-TN characterization results. 

### 3.2. Effect of Solution pH

**Figure 3:**
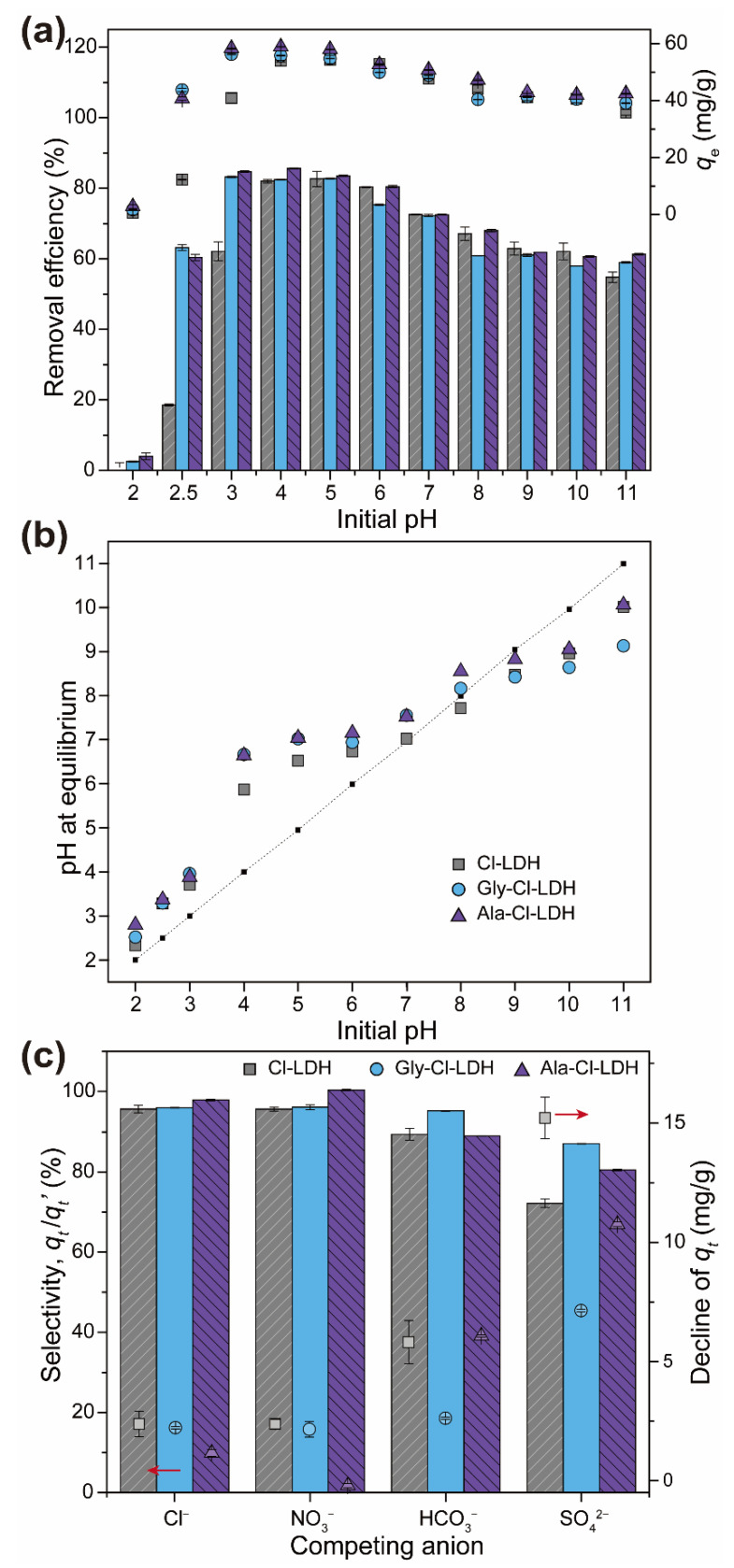
Effect of environmental factors on phosphate adsorption on Cl-LDH, Gly-Cl-LDH and Ala-Cl-LDH. Here, (**a**) represents phosphate removal and *q_e_* as a function of initial pH, (**b**) is the final pH of the adsorption system as a function of initial pH and (**c**) represents the effect of competing anions. (LDH dosage = 0.3 g/L, [Po] = 20 mg-P/L, retention time = 2 h).

The solution pH is one of the key influencing factors for phosphate adsorption onto LDH, which dynamically changes during the adsorption process. It not only affects the physicochemical properties of LDH, but also determines the distribution state and main valence of the adsorbate (phosphate), coexisting ions (e.g., carbonate) and the intercalated zwitterionic glycine (or alanine). The pH-related dissociation equilibria of phosphate, glycine and alanine are described in Section S3 of the Supplementary Materials. In the initial pH range of 2.0 to 11.0, the as-synthesized Cl-LDH, Gly-Cl-LDH and Ala-Cl-LDH exhibit a buffering effect on the solution (Figure 3b). Cl-LDH, Gly-Cl-LDH and Ala-Cl-LDH all show the best adsorption performance under acidic conditions (Figure 3a). The optimal initial pH ranges for the adsorption of phosphate by Cl-LDH, Gly-Cl-LDH and Ala-Cl-LDH are 3.0–5.0, 3.0–6.0 and 4.0–6.0, corresponding to the pH at the reaction equilibrium of 4.0–6.5, 4.0–7.0 and 6.0–7.0, respectively. We observed that Cl-LDH, Gly-Cl-LDH and Ala-Cl-LDH dissolved to varying degrees at low pHs (pH < 3.0); thus, the adsorption performances increased remarkably as the initial pH increased from 2.0 to 3.0. When the pH further increased to above 6.0, the removal of phosphate gradually decreased. 

According to the ionization state distribution of phosphate at different pH values, H_2_PO_4_^−^ is the main form of phosphate at a pH ranging from 3.0 to 6.0; thus, the ion exchange between Cl^−^ and phosphate is approximately equimolar. As the pH increases to above 6.0, phosphate transforms from the monovalent state to the divalent state, leading to a visible decrease in the phosphate (as element P) removal efficiency. In addition, with the increase in the pH, the surface of LDH would be deprotonated and carry more negative charges, leading to the increase in the competition for adsorption sites between phosphate and OH^−^ [37]. According to the determination of the zeta potential at different pH levels, the isoelectric point (IEP) of Cl-LDH is 11.76, and the IEP of Gly-Cl-LDH and Ala-Cl-LDH are above 12.0. Such results indicate that Cl-LDH, Gly-Cl-LDH and Ala-Cl-LDH have a constant positive charge in the experimental pH range. As the pH decreases, the zeta potential of LDH gradually increases, and the electrostatic interaction with phosphate increases accordingly [27]. In particular, Gly-Cl-LDH and Ala-Cl-LDH perform better than Cl-LDH at pH ≤ 7.0. When the pH < 6, the interlayer glycine and alanine exist in the form of zwitterionic ^−^OOC-R-NH_3_^+^, and carry more positive charges at a lower pH (Figure S3), facilitating the electrostatic interaction between phosphate and interlayer species. 

### 3.3. Effect of Coexisting Anions

We studied the effect of four prevalent coexisting anions (Cl^−^, NO_3_^−^, HCO_3_^−^ and SO_4_^2−^) on phosphate removal by Cl-LDH, Gly-Cl-LDH and Ala-Cl-LDH. In Figure 3c, the phosphate adsorption selectivity (%) means the molar ratio of the removed phosphate (mg/g) in the presence of the coexisting ion to the removed phosphate (mg/g) in the absence of the coexisting ion. The pH of the solution was set to near neutral to simulate the ionization state of coexisting ions in real wastewater. Figure 3c shows that the adsorption selectivity of phosphate by Cl-LDH, Gly-Cl-LDH and Ala-Cl-LDH remains dominant when any of the four competing anions coexists. The distribution coefficient (*K*_d_) of phosphate is greater than 10^3^ mL/g in the presence of Cl^−^, NO_3_^−^, HCO_3_^−^ and SO_4_^2−^, and even greater than 10^4^ mL/g in the presence of Cl^−^ and NO_3_^−^. Such results indicate that Cl-LDH, Gly-Cl-LDH and Ala-Cl-LDH are excellent adsorbents for phosphate [38]. The order of the inhibitory effect on the phosphate adsorption by Cl-LDH, Gly-Cl-LDH and Ala-Cl-LDH is SO_4_^2−^ > HCO_3_^−^ > Cl^−^ > NO_3_^−^, which is related to the charging of the anions [33,39]. The inhibitory effect of HCO_3_^−^, Cl^−^ and NO_3_^−^ is limited. Due to the correlation of the ionization state of anions and the pH, the coexistence of carbonate slightly inhibited the adsorption of phosphate, although CO_3_^2−^ has a high affinity with the hydroxide layers of LDH [40]. Over the course of adsorption, carbonate mainly existed as CO_2_ (aq) and HCO_3_^−^, while phosphate was mainly present as HPO_4_^2−^ and H_2_PO_4_^−^ (see Figures S1 and S2 in the supplementary material). CO_2_ (aq) can be degassed through agitation and centrifugation [11], and monovalent HCO_3_^−^ has a lower affinity with LDH than CO_3_^2−^ and phosphate. The inhibitory effect of SO_4_^2−^ was weaker when the adsorbents were Gly-Cl-LDH and Ala-Cl-LDH, which might be correlated to the forming of hydrogen bonds between phosphate and amidogen and carboxyl in the glycine and alanine molecule.

### 3.4. Effect of Contact Time and Adsorption Kinetics

**Figure 4:**
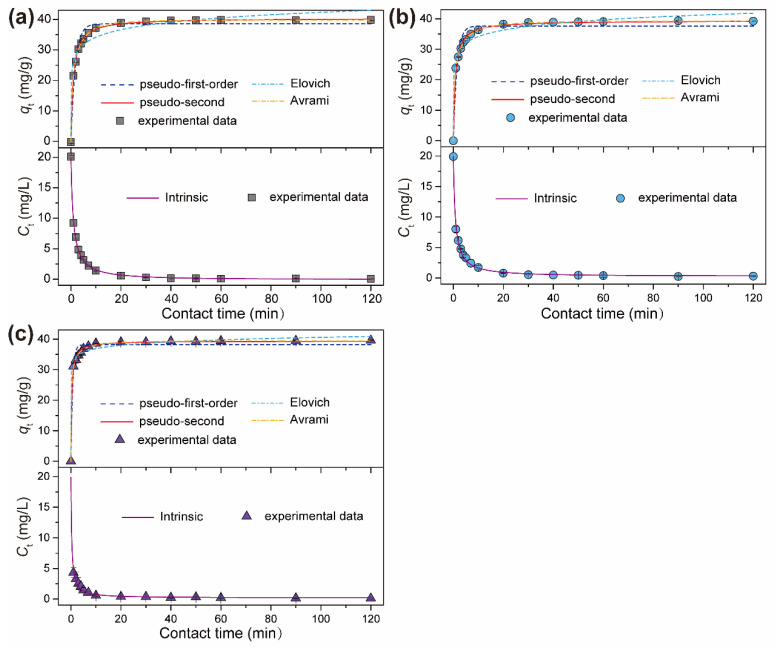
Adsorption kinetics and the fitting curves of pseudo-first, pseudo-second, Elovich, Avrami and intrinsic equations for phosphate adsorption onto Cl-LDH (**a**), Gly-Cl-LDH (**b**) and Ala-Cl-LDH (**c**). (LDH dosage = 0.5 g/L, [Po] = 20 mg-P/L, pH = 5.4).

The adsorption kinetics data were obtained by adding 0.5 g/L LDH into 100 mL of 20 mg/L phosphate solution, as presented in Figure 4. The dosage here was determined in the preliminary experiment regarding the effect of LDH dosage (Figure S6). The results showed that when the dosage continued to increase to above 0.5 g/L, the removal efficiency did not change significantly (paired sample *t*-test, *p* > 0.05), while the *q_e_* decreased significantly (paired sample *t*-test, *p* < 0.05). The data show that Cl-LDH, Gly-Cl-LDH and Ala-Cl-LDH remove phosphate rapidly at the beginning, and the removal efficiency reaches 51.9%, 58.0% and 78.1% in the first minute, respectively. At 20 min, the removal efficiencies of phosphate by Cl-LDH, Gly-Cl-LDH and Ala-Cl-LDH exceed 96%. After that, the adsorption slows down and tends to equilibrium at about 30 min. 

As shown in Figure 4 and Table 3, we employed five kinds of model (the pseudo-first model, pseudo-second model, Elovich model, Avrami model and intrinsic model) to nonlinearly fit the experimental data. Through the comparison of the coefficient of determination (R^2^) and the sum of errors squared (SSE), the pseudo-second model, Avrami model and intrinsic model all are evidently better. The pseudo-second-order kinetics model is widely applied in the description of chemisorption involving ion exchange [41]. The good fitting to the pseudo-second-order model also indicated that chemisorption was the main mechanism of phosphate adsorption by Cl-LDH, Gly-Cl-LDH and Ala-Cl-LDH.

Surprisingly, after fitting the kinetic data of the triplicated experiments to the kinetic models, we found that the rate constants (*k*_2_ in the pseudo-second model, *k* in the Avrami model and *k_int_* the intrinsic model) of Gly-Cl-LDH and Ala-Cl-LDH were significantly greater than that of Cl-LDH (paired sample *t*-test, *p* < 0.05), which means that the rate of phosphate adsorption by Cl-LDH evidently increased after being partially intercalated with glycine and alanine. The reason for the faster adsorption kinetics of Gly-Cl-LDH and Ala-Cl-LDH may be the enlargement of the interlayer space and the improvement of the surface structure, which is consistent with the findings of the XRD and the specific surface area and pore structure analysis. In addition, the electrostatic interaction between the intercalated glycine of alanine and phosphate may also accelerate the reaction, thereby increasing the adsorption rate. 

In the previous work, we developed the intrinsic kinetic model that introduces the influence of experimental initial conditions on the adsorption kinetics, and realized the direct comparison of the kinetic characteristics of different adsorbents under different initial conditions. We collected the kinetic results of recently studied phosphate adsorbents, and obtained the kinetic data using Plot Digitizer software. After fitting the experimental data with the intrinsic kinetic model, we extracted the fitting results with a corrected R^2^ value greater than 0.97. The data were screened according to the initial conditions of the kinetic experiment. The data in Table 4 were obtained from studies with similar initial conditions to this study (*ξ_ini_* is 0.58–0.77), which is more comparable. In Table 4, the kinetic constant *k_ini_* (representing the adsorption reaction rate) of MOF and LDH are 1~2 orders of magnitude larger than other phosphate adsorbents, while the *k_ini_* of LDH is larger than MOF. Therefore, compared with other phosphate adsorbents, LDH shows kinetic advantages, and the adsorption rate is further improved after glycine and alanine intercalation. Such findings are interesting because, despite the extensive attention on capacity improvement research, studies on the increase in the phosphate adsorption rate are limited, but of practical significance. The findings of this study can provide an effective method for improving the adsorption uptake rate of LDH adsorbents.

### 3.5. Adsorption Isotherms

**Figure 5:**
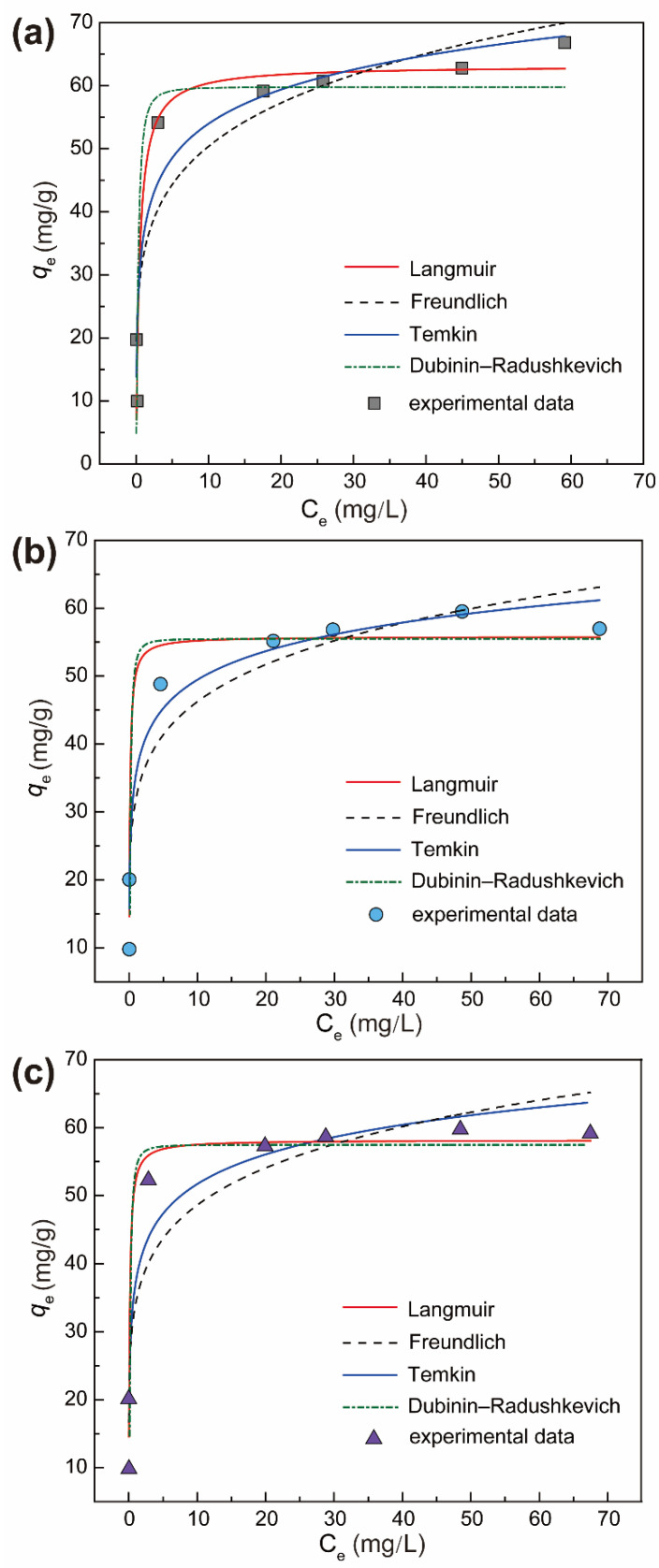
Adsorption isotherms and the fitting curves of Langmuir, Freundlich, Temkin and Dubinin–Radushkevich equations for phosphate adsorption onto Cl-LDH (**a**), Gly-Cl-LDH (**b**) and Ala-Cl-LDH (**c**). (LDH dosage = 0.3 g/L, [Po] = 20 mg-P/L, retention time = 2 h, pH = 6.50 ± 0.02).

As displayed in Figure 5, at 25 °C, the isotherms of phosphate adsorption by Cl-LDH, Gly-Cl-LDH and Ala-Cl-LDH are classified as typical H-type curve isotherms. H-type curves are usually observed in ion-exchange reactions [49]. In Figure 5, the *C_e_* (mg-P/L) and *q_e_* (mg-P/g) are the average value of the phosphate equilibrium concentration and the removal of phosphate at equilibrium of the triplicate experiments. We fitted the experimental data with Langmuir, Freundlich, Temkin and Dubinin–Radushkevich (D-R) isotherm models, and obtained the goodness of fit for each model (Table 5). The Langmuir isotherm model fits well with the two isotherms, indicating the homogeneous adsorption, with all the adsorption sites on the surface having equal affinity to the adsorbate, while the adsorption process involves the landing of monolayer phosphates on LDHs [50]. The estimated adsorption capacity *q_m_* (mg/g) of Cl-LDH, Gly-Cl-LDH and Ala-Cl-LDH are 63.2, 55.8 and 58.2 mg/g, exhibiting a good consistency with the experimental data. The phosphate adsorption capacity of Cl-LDH in this study is higher than most of the reported values [39,51,52,53,54,55]. The phosphate adsorption capacity of Gly-Cl-LDH and Ala-Cl-LDH is slightly lower than that of Cl-LDH (paired sample *t*-test, *p* < 0.05), which could be due to the partial substitution of ion-exchangeable chloride ions by the amino acids and the decrease in anion exchange capacity.

The dimensionless separation factor *r* is defined in Equation (1) [3,56]:

(1) $$r=\frac{1}{1+bC_{0}}$$

where *b* is the Langmuir constant (L/mg) and *C*_0_ is the initial phosphate concentration (mg/L). The calculated *r* values fall between 0 and 1, indicating the favorable and reversible adsorption of phosphate on Cl-LDH, Gly-Cl-LDH and Ala-Cl-LDH [57,58].

The phosphate adsorption capacities derived from the Langmuir models of other researched adsorbents are listed in Table 6. All the experiments were taken in artificial solutions using the studied adsorbents. It is worth noting that the direct comparison of phosphate adsorption capacities by various adsorbents is difficult due to the different adsorption conditions (e.g., pH, temperature, etc.) in different studies. We concluded from the salient generalization that the phosphate adsorption capacity of LDH is prominent and stable among the adsorbents. Despite the decline in the adsorption capacity after glycine and alanine intercalation, Gly-Cl-LDH and Ala-Cl-LDH still exhibit evident superiority over common phosphate adsorbents. In view of the significant increase in the adsorption rate and selectivity of LDH after glycine intercalation, the modification is still of significance.

### 3.6. Suggested Adsorption Mechanisms

**Figure 6:**
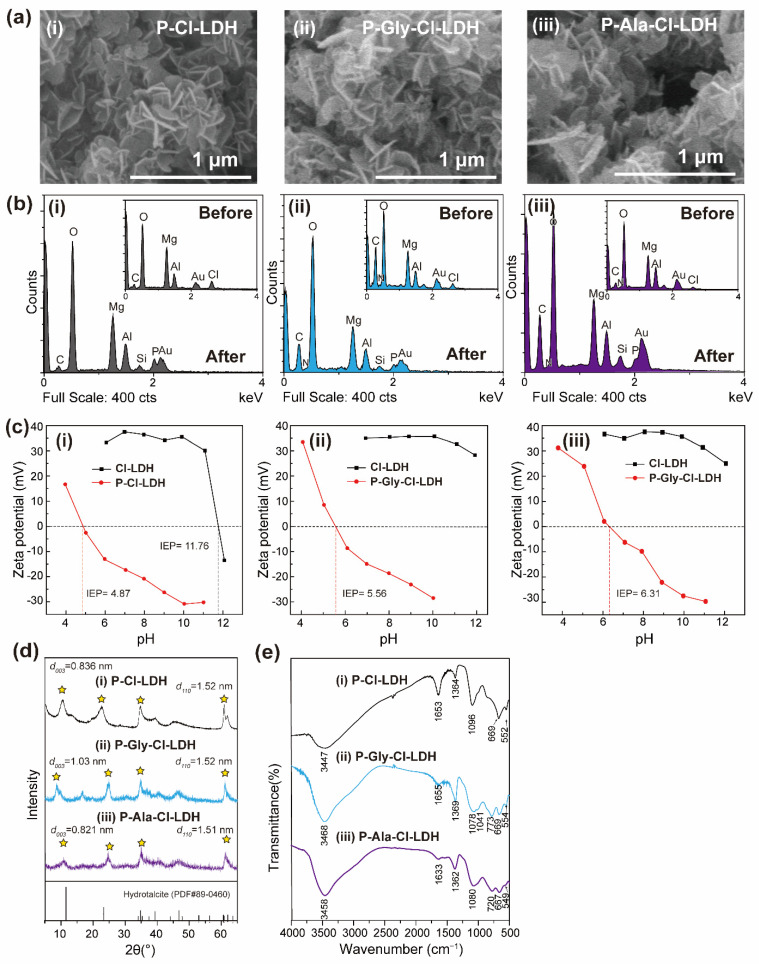
Characterization results of Cl-LDH (**i**), P-Gly-Cl-LDH (**ii**) and P-Ala-Cl-LDH (**iii**): (**a**) SEM images, (**b**) EDS spectra, (**c**) zeta potential as a function of pH, (**d**) XRD patterns (characteristic reflections are marked with stars), (**e**) FTIR spectra.

Figure 6 displays the characterization results of Cl-LDH, Gly-Cl-LDH and Ala-Cl-LDH after phosphate adsorption, in which the phosphate-adsorbed LDHs are named P-Cl-LDH, P-Gly-Cl-LDH and P-Ala-Cl-LDH. The morphology and characteristic structure of Cl-LDH, Gly-Cl-LDH and Ala-Cl-LDH remain after phosphate uptake, as shown in Figure 6a. The changes in the chemical composition before and after treatment (Figure 6b) reveal the mechanisms of the adsorption reaction. In Figure 6b, the element Cl disappears, and element P appears in the EDS spectra, demonstrating the anion exchange between chloride and phosphate. In the FTIR spectra (Figure 6e), bands at near 1096 cm^−1^ (Figure 6e(i)), 1078 cm^−1^ (Figure 6e(ii)) and 1080 cm^−1^ (Figure 6e(iii)) represent the P-O bending vibration, indicating the existing of phosphate in the LDH structure after adsorption. As presented in Figure 6b, the content of element C in P-Gly-Cl-LDH and P-Ala-Cl-LDH was observed to be less than that in Gly-Cl-LDH and Ala-Cl-LDH. Meanwhile, in Figure 6e, the vibration bands of alkyl C-H and amine N-H were hardly observed, and the asymmetric and symmetric stretching vibrations of R-COO^−^($\nu_{{R-\mathrm{COO}}^{-}}$) were identified at ~1369 and 1560 cm^−1^. Such results indicate that the glycine content in Gly-Cl-LDH and Ala-Cl-LDH decreased after phosphate adsorption. This may be due to the electrostatic repulsion between the positively charged amino group of glycine and the LDH laminate, and the intercalated glycine or alanine with lower charge densities could be exchanged by anions with a higher charge density (e.g., phosphate) and released into the aqueous solution. Such a phenomenon has also been seen in the previous study [68].

**Scheme 1:**
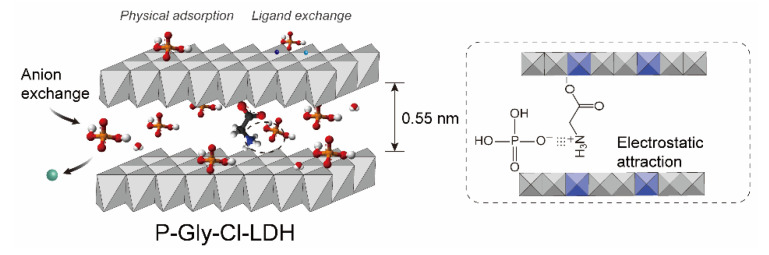
Schematic structural models of P-Gly-Cl-LDH and adsorption mechanisms.

The interlayer space of Cl-LDH, Gly-Cl-LDH and Ala-Cl-LDH show an increase after phosphate adsorption (Figure 6c), and the interlayer space of P-Cl-LDH is in accordance with Mg_2_Al_1_-Cl-LDH in other researches [69,70,71]. The interlayer space mainly depends on the charge, size and extent of the hydration of interlayer anions, the anion conformation, hydrogen bonds and layer charge density [55,71]. Since the size of phosphate ions is larger than that of chloride ions, the phenomenon of increased interlayer space after adsorption also confirms the occurrence of ion exchange [66,72]. It was also observed that the interlayer space of P-Gly-Cl-LDH was evidently larger than that of P-Cl-LDH and P-Ala-Cl-LDH. Meanwhile, the interlayer space of P-Gly-Cl-LDH was also larger than the longest axial length of the glycine molecule. Although the content of glycine was observed to decrease after phosphate adsorption by Gly-Cl-LDH, the increase in the interlayer space of P-Gly-Cl-LDH may be due to the charge balance caused by the electrostatic interaction between the positively charged amino group in zwitterionic glycine and phosphate, leading to the rearrangement of the remaining glycine (Scheme 1).

In Figure 6c, the isoelectric points (IEP) of P-Cl-LDH, P-Gly-Cl-LDH and P-Ala-Cl-LDH were 4.87, 5.56 and 6.31, respectively. The constant positive charge of Cl-LDH and Gly-Cl-LDH in the experimental pH range facilitated the electrostatic interaction with anions. The IEP of P-Cl-LDH, P-Gly-Cl-LDH and P-Ala-Cl-LDH exhibits a decrease after adsorption, which could be due to the decrease in the charge density on the adsorbent surface caused by the electrostatic attraction and ligand exchange with phosphate [45,55]. In addition, since LDH has an abundant pore structure, it also has a physical adsorption effect on ions in aqueous solutions.

As shown in Scheme 1, we deduced that the main mechanisms of phosphate adsorption by Cl-LDH, Gly-Cl-LDH and Ala-Cl-LDH are ion exchange and electrostatic adsorption, and ligand exchange and physical adsorption may also occur in the adsorption process. In particular, the amino part of glycine intercalated in Gly-Cl-LDH and phosphate may have an electrostatic interaction with phosphate.

## 4. Conclusions

In the present study, glycine was intercalated into LDH through the fast coprecipitation method, and Gly-Cl-LDH and Ala-Cl-LDH with partially intercalated glycine and alanine were successfully synthesized. The effect of glycine and alanine intercalation on the structure, chemical composition and phosphate adsorption properties of LDH were investigated through a comparative study of the pristine Cl-LDH, Gly-Cl-LDH and Ala-Cl-LDH. The characterization and adsorption experiments showed that the intercalated glycine and alanine changed the structure and improved the phosphorus removal performance, especially the kinetic properties. Results were found that showed that, compared with Cl-LDH, Gly-Cl-LDH and Ala-Cl-LDH have the higher porosity and larger specific surface area. The interlayer space increased after glycine or alanine intercalation. The optimum initial pH levels for the adsorption of phosphate by Cl-LDH, Gly-Cl-LDH and Ala-Cl-LDH are 3.0–5.0, 3.0–6.0 and 4.0–6.0. Phosphate adsorption by Cl-LDH and Gly-Cl-LDH remained dominant in the presence of the common anions Cl^−^, NO_3_^−^, HCO_3_^−^ and SO_4_^2−^, and Gly-Cl-LDH and Ala-Cl-LDH exhibited a better selectivity compared with Cl-LDH. The order of the influence on the phosphate adsorption by Cl-LDH and Gly-Cl-LDH is SO_4_^2−^ > HCO_3_^−^ > Cl^−^ > NO_3_^−^. The adsorption kinetics of phosphate adsorption onto Cl-LDH, Gly-Cl-LDH and Ala-Cl-LDH fit well with pseudo-second order model, and the rate constants of Gly-Cl-LDH and Ala-Cl-LDH are obviously higher than that of Cl-LDH, demonstrating an increase in the adsorption rate after intercalation. Compared with other phosphate adsorbents, the adsorption kinetics of Gly-Cl-LDH and Ala-Cl-LDH are advantageous. The phosphate adsorption capacities of Gly-Cl-LDH (55.8 mg/g) and Ala-Cl-LDH (58.2 mg/g) are slightly lower than that of Cl-LDH (63.2 mg/g). The suggested mechanisms of phosphate adsorption by Cl-LDH, Gly-Cl-LDH and Ala-Cl-LDH are ion exchange, electrostatic adsorption, ligand exchange and physical adsorption. Under the most optimal opreation conditions (25 °C, pH = 5.4, C_0_ = 20 mg-P/L, dose = 0.5 mg/L), the removal efficiency of Gly-Cl-LDH and Ala-Cl-LDH can reach 58% and 78.1% at the first minute and reach > 99.5 at 2 h. The findings in this study indicated that glycine- and alanine-intercalated LDH demonstrated considerable potential for their future practical application in phosphate removal in terms of the improvement in the adsorption rate.

## Figures and Tables

**Table 1:** Chemical compositions of Cl-LDH, Gly-Cl-LDH and Ala-Cl-LDH.

Sample	Metal Ratio in Precursor	Determined Chemical Composition	Wt %, Found (calcd.)			
			Mg	Al	C	N
Cl-LDH	Mg_0.67_Al_0.33_	Mg_0.67_Al_0.33_(OH)_2_Cl_0.33_·0.28H_2_O	18.1 ± 0.13	9.80 ± 0.06	-	-
			(21.4)	(11.7)	-	-
Gly-Cl-LDH	Mg_0.67_Al_0.33_	Mg_0.67_Al_0.33_(OH)_2_Gly_0.03_Cl_0.30_·0.28H_2_O	17.5 ± 0.08	9.60 ± 0.10	1.14 ± 0.03	0.55 ± 0.01
			(21.4)	(11.7)	(0.95)	(0.55)
Ala-Cl-LDH	Mg_0.67_Al_0.33_	Mg_0.67_Al_0.33_(OH)_2_Ala_0.03_Cl_0.30_·0.21H_2_O	17.6 ± 0.10	9.60 ± 0.06	1.46 ± 0.05	0.55 ± 0.01
			(22.0)	(11.4)	(1.43)	(0.56)

**Table 2:** PXRD lattice constants of Cl-LDH, Gly-Cl-LDH and Ala-Cl-LDH.

Sample	(003) (°)	*d*_003_ (Å)	Interlayer Space (Å)	(110) (°)	*d*_110_ (Å)	*a* (Å)
Cl-LDH	11.69	7.57	2.77	61.17	1.52	3.03
Gly-Cl-LDH	11.48	7.71	2.91	60.86	1.52	3.05
Ala-Cl-LDH	11.37	7.78	2.98	60.82	1.52	3.05

**Table 3:** Adsorption kinetic parameters and correlation coefficients of phosphate adsorption by Cl-LDH, Gly-Cl-LDH and Ala-Cl-LDH.

Kinetics Model		Cl-LDH	Gly-Cl-LDH	Ala-Cl-LDH
Pseudo-first-order model	*q_e_* (mg/g)	38.6	37.6	38.2
	*k*_1_	0.565	0.683	1.444
	R^2^	0.967	0.952	0.973
	RSS	53.061	70.959	37.262
Pseudo-second-order model	*q_e_* (mg/g)	40.4	39.4	39.4
	*k*_2_	0.025	0.032	0.080
	R^2^	0.998	0.996	0.997
	RSS	2.876	6.235	4.151
Elovich model	*α* (mg·g^−1^)	5.54 × 10^3^	1.99 × 10^4^	1.28 × 10^9^
	*β* (mg·g^−1^)	0.283	0.324	0.618
	R^2^	0.965	0.973	0.989
	RSS	0.958	39.801	15.574
Avrami model	*q_e_* (mg/g)	39.7	39.3	39.5
	*k*	0.766	0.910	1.502
	*n*	0.549	0.452	0.342
	R^2^	1.000	1.000	0.999
	RSS	0.702	0.502	1.142
Intrinsic model	*ξ_eq_*	0.971	1.016	1.012
	*k_int_*	3.368	4.102	10.170
	R^2^	0.998	0.996	0.997
	RSS	0.724	1.606	1.095
	*ξ_ini_*	0.636	0.713	0.683

**Table 4:** Intrinsic kinetic parameters of phosphate adsorption materials.

Material Type	Adsorbent	Parameters of Intrinsic Model				Reference
		*ξ_ini_*	*k_int_*	*ρ*	*ξ_eq_*	
Metal oxide and hydroxide (excluding LDH)	Fe_1_–Cu_2_ binary oxide	0.71	0.561	0.2	1.32	[42]
	Fe_6_–Mn_1_ binary oxide	0.75	0.303	0.2	1.15	[43]
	Iron oxide	0.66	0.120	0.6	1.05	[44]
	HMO@NS	0.71	0.041	0.5	0.29	[45]
Functionalized mesoporous silica	La_25_M41	0.74	0.002	1.0	2.49	[46]
Industrial by-products	P-CSH	0.68	0.006	5.0	1.33	[6]
Modified clay minerals	La-Z	0.58	0.016	2.0	1.02	[47]
Metal organic framework	0.75Ce-UiO-66-NH_2_	0.59	1.340	0.4	1.05	[10]
	Ce-BDC	0.61	1.385	0.5	1.00	[9]
Layered double hydroxides (LDH)	LDHns-U25	0.51	1.773	1.0	1.16	[48]
	Cl-LDH	0.67	3.368	0.5	0.97	This work
	Gly-Cl-LDH	0.71	4.102	0.5	1.02	
	Ala-Cl-LDH	0.68	10.17	0.5	1.01	

**Table 5:** Adsorption isotherm parameters and correlation coefficients of phosphate adsorption by Cl-LDH and Gly-Cl-LDH.

LDH Type	Langmuir			Freundlich			Temkin			Dubinin–Radushkevish		
	*q_m_* ^1^	*B* ^2^	R^2^	*K_F_* ^3^	*n*	R^2^	*K_t_*	*B*	R^2^	*q_m_* ^1^	*B_D_* ^4^	R^2^
Cl-LDH	63.2	2.16	0.930	32.90	5.42	0.871	100.11	7.81	0.914	59.8	0.05	0.876
Gly-Cl-LDH	55.8	9.13	0.958	31.92	6.21	0.918	347.15	6.07	0.962	55.4	0.02	0.954
Ala-Cl-LDH	58.2	8.60	0.973	34.10	6.50	0.881	368.51	6.30	0.942	57.7	0.02	0.968

^1^ *q_m_* in mg/g. ^2^ *b* in L/mg. ^3^ *K_F_* in (mg/g)/(mg/L)*^n^*. ^4^ *B_D_* in mol^2^/kJ^2^.

**Table 6:** Comparison of phosphate adsorption capacities of various adsorbents in artificial solutions.

Adsorbent	Specific Surface Area (m^2^/g)	*q*_max_^1^ (mg-P/g)	pH	Equilibrium Time (h)	Temperature (°C)	Reference
Aluminum salt slag	16.7	2.3–3.5	7.0	48	25	[59]
Composite metal oxides	307.2	26.3	6.0	24	25	[60]
Iron oxide tailings	47.9	8.2	6.6	>24	20–21	[58]
Bentonite	85.0	0.37	5.45	24	25	[4]
Kaolinite	3.7	0.62	5.45	-	25	
Zeolite	13.8	0.63	5.45	-	25	
Bauxite	6.8	0.61	-	24	21	[5]
La/Al-pillared bentonite		13	5.0	12	25	[61]
Alkaline and La-modified zeolite	-	9.1	7.0	4	39.85	[62]
Purolite FerrIX A33E resin		48.0	7.2–7.6	48	24	[63]
Sponge iron	≥80	1.1	-	8	25	[64]
Zn-Al-CO_3_-LDH	135	68.4	-	1	25	[51]
Mg-Al-CO_3_-LDH	104	31.3	-	1	25	
Calcined Mg-Cl-NO_3_-LDH	210.0	44.0	6.0	4	29.85	[33]
Mg-Al-NO_3_-LDH	5.7	64.1	6.0	2	20	[65]
Zn-Al-PMA-Cl-LDH	-	57.1	-	4	20	[18]
Calcined Mg-Fe-Cl-LDH	-	9.8	7.0	2	25	[66]
Zn-Al- NO_3_-LDH	135	68.4	-	0.67	25	[51]
Mg-Al-NO_3_-LDH	104	31.3	-	1	25	
Zn- Fe-Zr-Cl-LDH	115	20.3	7.0–8.0	1	25	[67]
Cl-LDH	13.4	63.2	5.5	0.5	25	This study
Gly-Cl-LDH	19.0	55.8	5.5	0.5	25	
Ala-Cl-LDH	21.5	58.2	5.5	0.5	25	

^1^ *q*_max_ is the maximum phosphate adsorption capacity calculated from the Langmuir equation.

## Data Availability

Data can be available upon request from the authors.

## Supplementary Materials

The following supporting information can be downloaded at: https://www.mdpi.com/article/10.3390/nano12040586/s1, Section S1: Description of parameters reflecting the phosphate adsorption performance, Section S2: Description of kinetics and isotherm fitting models, Section S3: Description of the theoretical calculations of pH-related dissociation equilibria of phosphate, carbonate and glycine; Figure S1:Distribution curves of different phosphate state within the pH of 0~14, Figure S2: Distribution curves of different carbonate state within the pH of 0~14, Figure S3: Distribution curves of different glycine (a) and alanine (b) state within the pH of 0~14, Figure S4: Particle size distribution Cl-LDH, Gly-Cl-LDH and Ala-Cl-LDH, Figure S5: N_2_ adsorption-desorption isotherms of Cl-LDH (e) and Gly-Cl-LDH (f), the inset is the pore size distribution determined by the BJH method, Figure S6: Effect of LDH dosage on phosphate adsorption on Cl-LDH, Gly-Cl-LDH and Ala-Cl-LDH. (a) represents phosphate concentration in supernatant as a function of dosage, (b−d) are the phosphate removal (%) and *q_e_* (mg/g) as a function of pH; Table S1: Particle size distribution Cl-LDH, Gly-Cl-LDH and Ala-Cl-LDH [41,56,73,74,75,76,77,78,79,80,81].
