# Supplementary material for: Glycine- and Alanine-Intercalated Layered Double Hydroxides as Highly Efficient Adsorbents for Phosphate with Kinetic Advantages
Source: Nanomaterials (Basel). 2022 Feb 9;12(4):586. doi: 10.3390/nano12040586 (PMC8878144; doi:10.3390/nano12040586)
Supplement: Supplementary file 1 [file nanomaterials-12-00586-s001.zip › nanomaterials-1542596-supplementary.pdf]

nanomaterials

Supporting Material

Glycine- and Alanine-Intercalated Layered Double Hydroxides as Highly Efficient Adsorbents for Phosphate with Kinetic Advantages

Qian Zhang, Fangying Ji *, Lei Jiang, Qiushi Shen, Yuanxiang Mao and Caocong Liu

College of Environment and Ecology, Chongqing University, Chongqing 400045, China; zhangqiancqu@cqu.edu.cn (Q.Z.); jginger067@gmail.com (L.J.); m15025439561@126.com (Q.S.); 20191701572@cqu.edu.cn (Y.M.); 20201701029@cqu.edu.cn (C.L.)

* Correspondence: jfy@cqu.edu.cn

Section S1. Description of parameters reflecting the phosphate adsorption performance

The uptake of phosphate at equilibrium (q_e, mg/g) (Equtaion (S1)), phosphate removal efficiency (R) (Equation (S2)) and the distribution coefficient (K_d, mL/g) (Equation (S3)) for the adsorbent were calculated from the following equations:

$$q_e = \frac{(C_0 - C_e)V}{m} \tag{S1}$$

$$R(\%) = \frac{C_0 - C_e}{C_0} \times 100 \tag{S2}$$

$$K_d = \frac{(C_0 - C_t)V}{C_t \times m} \tag{S3}$$

where C_0, C_e and C_t is the concentration of phosphate as element P (mg/L) at initial, equilibrium and time t (min), V is the volume of the solution (in L for q_e calculation and in mL for K_d calculation) and m is the mass of adsorbents (dry weight, g). Our experiments were conducted with V/m ratios of 1666.7-10000 mL/g at ambient temperature.

Section S2. Description of kinetics and isotherm fitting models

a) Kinetics fitting models

Pseudo-first-order (Equation (S4))[1], pseudo-second-order (Equation (S5)) [2], Elovich (Equation (S6)) [3], Avrami [4] (Equation (S7)) and Intrinsic [5] (Equation (S8)) kinetics equation.

$$q_t = q_e(1 - e^{-k_1 t}) \tag{S4}$$

$$q_t = \frac{k_2 q_e^{\ 2} t}{1 + k_2 q_e t} \tag{S5}$$

$$q_t = a + b \ln t \tag{S6}$$

$$q_t = q_e \cdot (1 - e^{-kt^n}) \tag{S7}$$

$$C_t = \frac{c_0 \cdot (1 - \zeta_{eq})}{e^{k_{ini} \cdot \rho \cdot (1 - \zeta_{eq}) \cdot t} - \zeta_{eq}} \tag{S8}$$

where q_t and q_e (mg/g) are the amount of phosphate adsorbed at time t (min) and at equilibrium. k_1 (min^{-1}), k_2 (g/(mg·min)), k and k_{ini} ($L \cdot g^{-1} \cdot min^{-1}$) are the rate constant of the pseudo-first-order, pseudo-second-order, Avrami and intrinsic kinetics models, respectively. a is the initial adsorption rate (mg/g), and b is the desorption constant (mg/g). c_0 (mg/L) is the initial phosphate concentration, ρ (g/L) is the dosage of adsorbent. ξ_{eq} is the state constant at equilibrium.

b) Isotherm fitting models

Langmuir (Equation (S9)) [6], Freundlich (Equation (S10)) [7], Temkin (Equation (S11)) [8] Dubinin-Radushkevich (D-R) (Equations (S12) and (S13)) [9,10] isotherm model.

$$q_e = \frac{b q_m C_e}{1 + b C_e} \tag{S9}$$

$$q_e = k C_e^{\frac{1}{n}} \tag{S10}$$

$$q_e = A + B \ln C_e \tag{S11}$$

$$q_e = q_m \exp(-B_D \varepsilon_D^2) \tag{S12}$$

$$\varepsilon_D = RT \ln(1 + \frac{1}{C_e}) \tag{S13}$$

where q_e (mg/g) and C_e (mg/L) are the amount of phosphate adsorbed per g of adsorbent and the corresponding phosphate concentration in solution at the equilibrium. q_m (mg/g) is the maximum adsorption capacity. b (L/mg) and k ((mg/g)/(mg/L)^n) are the Langmuir and Freundlich adsorption equilibrium constant. A and B are isotherm constants in the Temkin isotherm equation which can be determined by regression of the experimental data [11].

c) Error function

The sum of the errors squared (SSE) was the objective error function in the optimization procedure.

$$\mathrm{SSE} = \sum (q_{\exp,i} - q_{cal,i})^2 \tag{S14}$$

where q_{exp} and q_{cal} are the experimental and model-predicted value, respectively.

Section S3. Description of the Theoretical Calculations of pH-Related Dissociation Equilibria of Phosphate, Carbonate and Glycine

a) The pH-Related Dissociation Equilibria of Phosphate

Phosphate has dissociation equilibrium in aqueous solutions of different pH as follows:

$$H_3PO_4 \rightleftharpoons H_2PO_4^- + H^+ \qquad K_1 = \frac{[H_2PO_4^-]\times[H^+]}{[H_3PO_4]} = 7.08\times10^{-3} \tag{S15}$$

$$H_2PO_4 \rightleftharpoons HPO_4^{2-} + H^+ \qquad K_2 = \frac{[HPO_4^{2-}]\times[H^+]}{[H_2PO_4^-]} = 6.31\times10^{-8} \tag{S16}$$

$$HPO_4 \rightleftharpoons PO_4^{3-} + H^+ \qquad K_3 = \frac{[PO_4^{3-}]\times[H^+]}{[HPO_4^{2-}]} = 5.01\times10^{-13} \tag{S17}$$

where $[H^+]$, $[H_3PO_4]$, $[H_2PO_4^-]$, $[HPO_4^{2-}]$ and $[PO_4^{3-}]$ are the molar concentrations of H^+, H_3PO_4, $H_2PO_4^-$, HPO_4^{2-} and PO_4^{3-}. $[H^+]$ could be calculated from pH value of the solution through: $[H^+] = 10^{-pH}$.

The ionization fraction α represents the fraction of a component in total concentration, which could be defined as Equation (18) (taking H_3PO_4 as an example).

$$\begin{aligned}\alpha_0 &= \frac{[H_3PO_4]}{[H_3PO_4]+[H_2PO_4^-]+[HPO_4^{2-}]+[PO_4^{3-}]} \\ &= \frac{1}{1+\frac{[H_2PO_4^-]}{[H_3PO_4]}+\frac{[HPO_4^{2-}]}{[H_3PO_4]}+\frac{[PO_4^{3-}]}{[H_3PO_4]}} \\ &= \frac{1}{1+\frac{K_1}{[H^+]}+\frac{K_1\times K_2}{[H^+]^2}+\frac{K_1\times K_2\times K_3}{[H^+]^3}} \\ &= \frac{[H^+]^3}{[H^+]^3+K_1\times[H^+]^2+K_1\times K_2\times[H^+]+K_1\times K_2\times K_3}\end{aligned} \quad (S18)$$

Similarly, the ionization fraction of α_1, α_2, α_3 for $H_2PO_4^-$, HPO_4^{2-} and PO_4^{3-} could be calculated through Equations (S19)–(S21).

$$\alpha_1 = \frac{K_1[H^+]^2}{[H^+]^3+K_1\times[H^+]^2+K_1\times K_2\times[H^+]+K_1\times K_2\times K_3} \quad (S19)$$

$$\alpha_2 = \frac{K_1K_2[H^+]}{[H^+]^3+K_1\times[H^+]^2+K_1\times K_2\times[H^+]+K_1\times K_2\times K_3} \quad (S20)$$

$$\alpha_3 = \frac{K_1K_2K_3}{[H^+]^3+K_1\times[H^+]^2+K_1\times K_2\times[H^+]+K_1\times K_2\times K_3} \quad (S21)$$

Within pH range of 1 to 14, the distribution curves of phosphate in different ionization state were calculated and plotted in Figure S1.

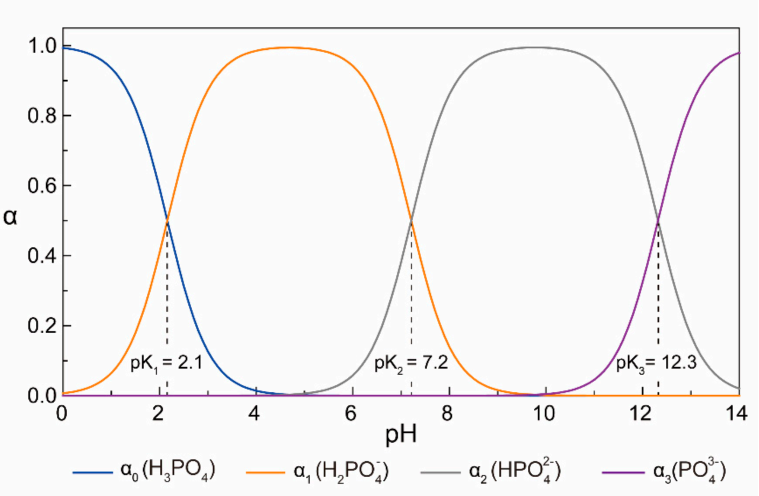

Figure S1. Distribution curves of different phosphate state within the pH of 0~14.

b) The pH-related Dissociation Equilibria of Carbonate

Carbonate has dissociation equilibrium in a closed system as follows (25 °C):

$$CO_2(aq)+H_2O \overset{K_m}{\rightleftharpoons} H_2CO_3 \qquad K_m = \frac{[H_2CO_3]}{[CO_2(aq)]} = 10^{-2.8} \tag{S22}$$

$$H_2CO_3 \overset{K_1}{\rightleftharpoons} HCO_3^- + H^+ \qquad K_1 = \frac{[HCO_3^-]\times[H^+]}{[H_2CO_3]} = 10^{-3.5} \tag{S23}$$

$$HCO_3^- \overset{K_2}{\rightleftharpoons} CO_3^{2-} + H^+ \qquad K_2 = \frac{[CO_3^{2-}]\times[H^+]}{[HCO_3^-]} = 10^{-10.3} \tag{S24}$$

where $[H^+]$, $[H_2CO_3]$, $[HCO_3^-]$ and $[CO_3^{2-}]$ are the molar concentrations of H^+, H_2CO_3, HCO_3^- and CO_3^{2-}. $[H^+]$ could be calculated from pH value of the solution through: $[H^+] = 10^{-pH}$.

The ionization fraction α represents the fraction of a component in total concentration, which could be defined as Equations (S25)–(S28).

$$\alpha_{CO_2(aq)} = \frac{[H^+]^2}{K_m \times ([H^+]^2 + K_1 \times [H^+] + K_1 \times K_2)} \tag{S25}$$

$$\alpha_{H_2CO_3} = \frac{[H^+]^2}{[H^+]^2 + K_1 \times [H^+] + K_1 \times K_2} \tag{S26}$$

$$\alpha_{HCO_3^-} = \frac{K_1[H^+]}{[H^+]^2 + K_1 \times [H^+] + K_1 \times K_2} \tag{S27}$$

$$\alpha_{CO_3^{2-}} = \frac{K_1 K_2}{[H^+]^2 + K_1 \times [H^+] + K_1 \times K_2} \tag{S28}$$

Within pH range of 1 to 14, the distribution curves of carbonate in different ionization state were calculated and plotted in Figure S2.

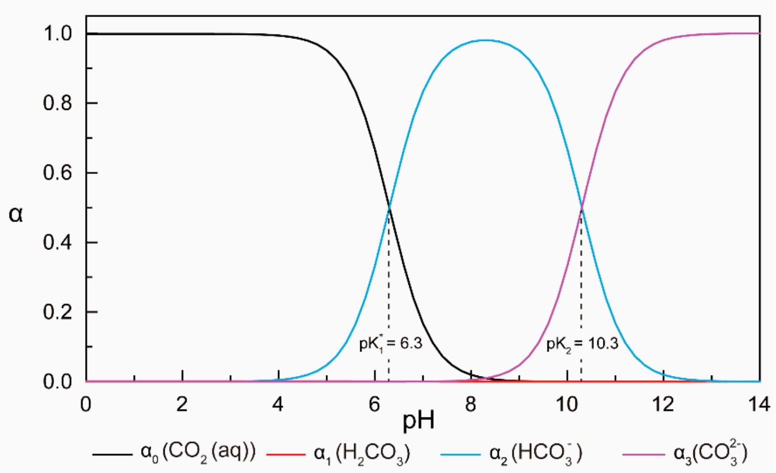

Figure S2. Distribution curves of different carbonate state within the pH of 0~14.

c) The pH-Related Dissociation Equilibria of Glycine and Alanine

Glycine and alanine undergoes internal acid-base reactions and exists in the form of internal salts known as zwitterions due to the acidity of carboxyl group and alkalinity of amino group. Since glycine mostly presents in the aqueous solution as zwitterions, which is much more than the the molecular form, the dissociation process of glycine and alanine in aqueous solutions can be briefly described as follows.

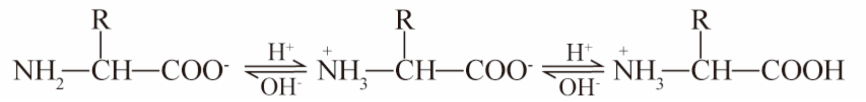

(S29)

The dissociation process of glycine and alanine in water is similar to that of a diacid, which could be described as follows (where H_2A represents the acid form with positive charge, HA represents the zwitterionic form, and A^- represents the basic form with negative charge):

$$H_2A^+ \overset{K_1}{\rightleftharpoons} HA + H^+ \quad K_1 = \frac{[HA]\times[H^+]}{[H_2A^+]} \quad pK_1\,(\text{glycine}) = pK_1\,(\text{alanine}) = 2.34 \tag{S30}$$

$$HA \overset{K_2}{\rightleftharpoons} A^- + H^+ \quad K_2 = \frac{[A^-]\times[H^+]}{[HA]} \quad pK_2\,(\text{glycine}) = 9.60;\ pK_2\,(\text{alanine}) = 9.69 \tag{S31}$$

where $[H^+]$, $[H_2A^+]$, [HA] and $[A^-]$ are the molar concentrations of H^+, H_2A^+, HA and A^-. $[H^+]$ could be calculated from pH value of the solution through: $[H^+] = 10^{-pH}$.

The ionization fraction α represents the fraction of a component in total concentration, which could be defined as Equations (S32)–(S34).

$$\alpha_{H_2A^+} = \frac{[H^+]^2}{[H^+]^2 + K_1\times[H^+] + K_1\times K_2} \tag{S32}$$

$$\alpha_{\mathrm{HA}} = \frac{K_1[\mathrm{H}^+]}{[\mathrm{H}^+]^2 + K_1 \times [\mathrm{H}^+] + K_1 \times K_2} \quad \text{(S33)}$$

$$\alpha_{\mathrm{A}^-} = \frac{K_1 K_2}{[\mathrm{H}^+]^2 + K_1 \times [\mathrm{H}^+] + K_1 \times K_2} \quad \text{(S34)}$$

Within pH range of 1 to 14, the distribution curves of glycine and alanine in different ionization state were calculated and plotted in Figure S3.

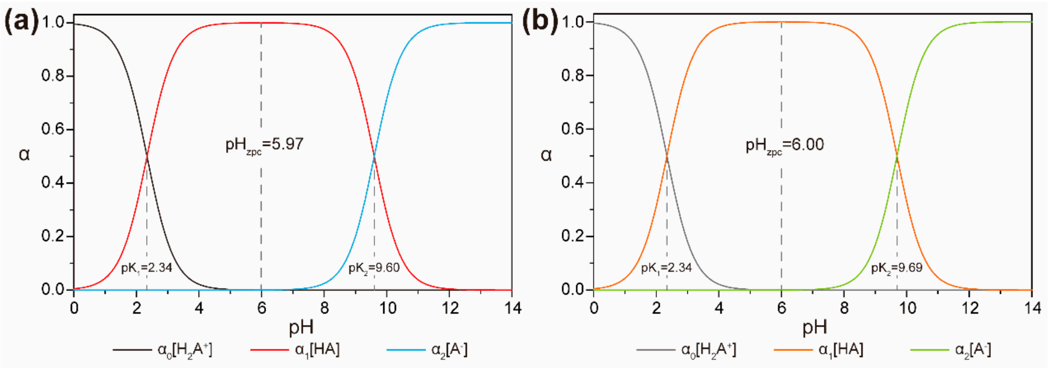

Figure S3. Distribution curves of different glycine (a) and alanine (b) state within the pH of 0~14.

Section S4.

Metal ions were measured by ICP-OES. For element Mg, the limit of detection (LOD) is 0.02 mg/L, and the limit of quantification (LOQ) is 0.09 mg/L. For element Al, the LOD is 0.009 mg/L, and the LOQ is 0.04 mg/L. TOC and TN was measured using a TOC analyzer equipped with a TN unit based on combustion oxidation-non-dispersive infrared absorption method. The LOQ of TOC is 4 μg/L, and the LOQ of TN is 0.1 mg/L. Phosphate concentration determination was performed on a UV-Vis spectrophotometer using the Molybdenum Blue method. The LOD is 0.01 mg/L (as element P), and the LOQ is 0.04

Table S1. Particle size distribution Cl-LDH, Gly-Cl-LDH and Ala-Cl-LDH.

Sample	d(0.1) (μm)	d(0.5) (μm)	d(0.9) (μm)
Cl-LDH	1.392	30.956	110.901
Gly-Cl-LDH	1.687	30.634	94.653
Ala-Cl-LDH	1.226	27.306	84.487

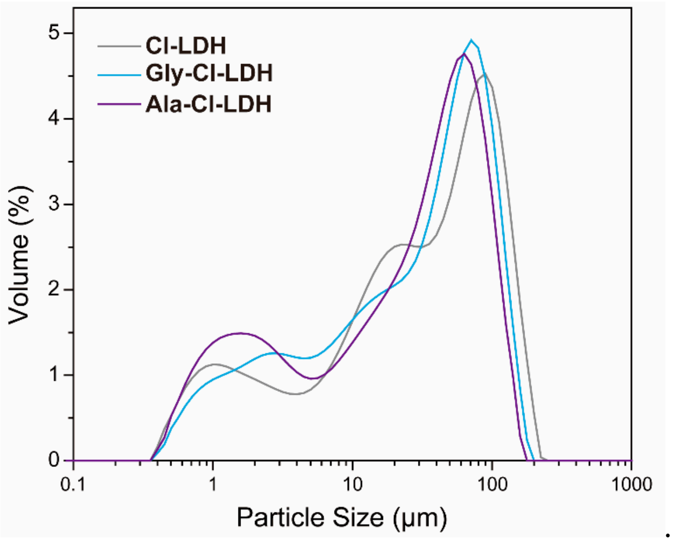

Figure S4. Particle size distribution Cl-LDH, Gly-Cl-LDH and Ala-Cl-LDH.

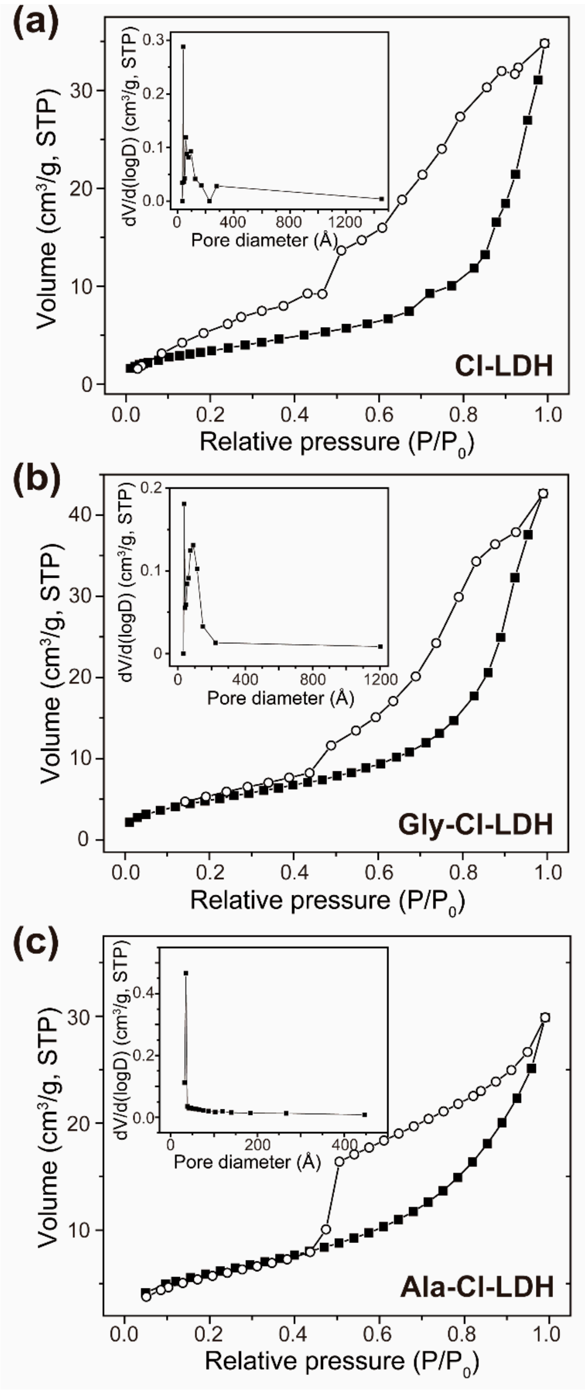

Figure S5. N_2 adsorption-desorption isotherms of Cl-LDH (e) and Gly-Cl-LDH (f), the inset is the pore size distribution determined by the BJH method.

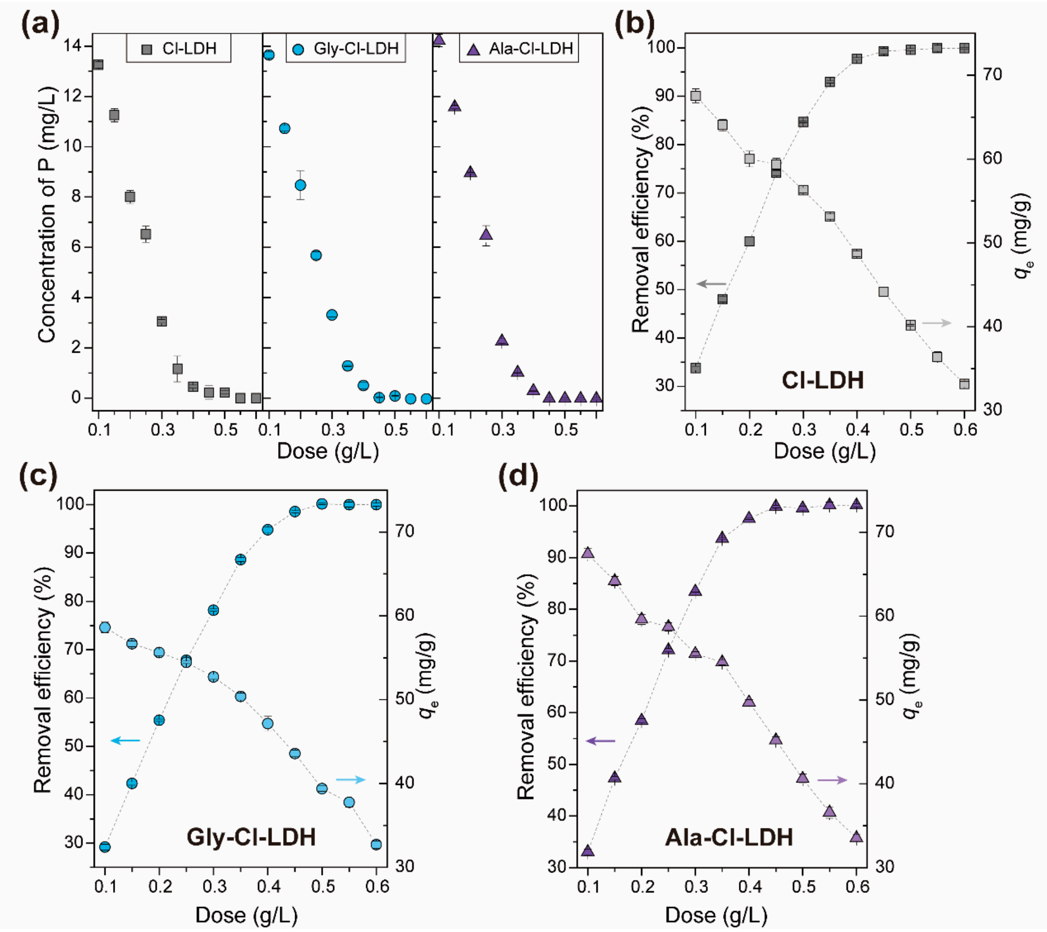

Figure S6. Effect of LDH dosage on phosphate adsorption on Cl-LDH, Gly-Cl-LDH and Ala-Cl-LDH. (a) represents phosphate concentration in supernatant as a function of dosage, (b)~(d) are the phosphate removal (%) and q_e (mg/g) as a function ofdosage. (LDH dosage = 0.1~0.6 g/L, [P_o] = 20 mg-P/L, retention time = 2 h). Error bars show the standard deviation (n = 3).

References

1. Lagergren, S. Zur theorie der sogenannten adsorption geloster stoffe. *Handlingar* **1898**, *24*, 1–39.
2. Ho, Y.-S. Review of second-order models for adsorption systems. *J. Hazard. Mater.* **2006**, *136*, 681–689.
3. Chien, S.H.; Clayton, W.R. Application of Elovich equation to the kinetics of phosphate release and sorption in soils 1. *Soil Sci. Soc. Am. J.* **1980**, *44*, 265–268.
4. Avrami, M. Kinetics of phase change. II Transformation-time relations for random distribution of nuclei. *J. Chem. Phys.* **1940**, *8*, 212–224.
5. Fang, D.; Zhuang, X.; Huang, L.; Zhang, Q.; Shen, Q.; Jiang, L.; Xu, X.; Ji, F. Developing the new kinetics model based on the adsorption process: From fitting to comparison and prediction. *Sci. Total Environ.* **2020**, *725*, 138490.
6. Langmuir, I. The constitution and fundamental properties of solids and liquids. Part I. Solids. *J. Am. Chem. Soc.* **1916**, *38*, 2221–2295.
7. Freundlich, H. Über die adsorption in lösungen. *Z. Phys. Chem.* **1907**, *57*, 385–470.
8. Temkin, M.I. Adsorption equilibrium and the kinetics of processes on nonhomogeneous surfaces and in the interaction between adsorbed molecules. *Zh. Fiz. Chim.* **1941**, *15*, 296–332.
9. Dubinin, M.M. The Potential Theory of Adsorption of Gases and Vapors for Adsorbents with Energetically Nonuniform Surfaces. *Chem. Rev.* **1960**, *60*, 235–241.
10. Vijayaraghavan, K.; Padmesh T.V.N.; Palanivelu, K.; Velan, M. Biosorption of nickel(II) ions onto Sargassum wightii: Application of two-parameter and three-parameter isotherm models. *J. Hazard. Mater.* **2006**, 133, 304–308.
11. Weber, T.W.; Chakravorti, R.K.; Pore and solid diffusion models for fixed-bed adsorbers. *Aiche J.* **1974**, *20*, 228–238.
